# Twelve-month outcomes of a randomized trial of a moderate-carbohydrate versus very low-carbohydrate diet in overweight adults with type 2 diabetes mellitus or prediabetes

**DOI:** 10.1038/s41387-017-0006-9

**Published:** 2017-12-21

**Authors:** Laura R. Saslow, Jennifer J. Daubenmier, Judith T. Moskowitz, Sarah Kim, Elizabeth J. Murphy, Stephen D. Phinney, Robert Ploutz-Snyder, Veronica Goldman, Rachel M. Cox, Ashley E. Mason, Patricia Moran, Frederick M. Hecht

**Affiliations:** 1 University of Michigan, Ann Arbor, MI USA; 2 San Francisco State University, San Francisco, CA USA; 3 Northwestern University, Evanston, IL USA; 4 University of California, San Francisco, CA USA; 5 Virta Health, San Francisco, CA 94105 USA; 6 UCSF Benioff Children’s Hospital Oakland, Oakland, CA USA

## Abstract

Dietary treatment is important in management of type 2 diabetes or prediabetes, but uncertainty exists about the optimal diet. We randomized adults (*n* = 34) with glycated hemoglobin (HbA1c) > 6.0% and elevated body weight (BMI > 25) to a very low-carbohydrate ketogenic (LCK) diet (*n* = 16) or a moderate-carbohydrate, calorie-restricted, low-fat (MCCR) diet (*n* = 18). All participa*n*ts were encouraged to be physically active, get sufficient sleep, and practice behavioral adherence strategies based on positive affect and mindful eating. At 12 months, participants in the LCK group had greater reductions in HbA1c levels (estimated marginal mean (EMM) at baseline = 6.6%, at 12 mos = 6.1%) than participants in MCCR group (EMM at baseline = 6.9%, at 12 mos = 6.7%), *p* = .007. Participants in the LCK group lost more weight (EMM at baseline = 99.9 kg, at 12 mos = 92.0 kg) than participants in the MCCR group (EMM at baseline = 97.5 kg, at 12 mos = 95.8 kg), *p* < .001. The LCK participants experienced larger reductions in diabetes-related medication use; of participants who took sulfonylureas or dipeptidyl peptidase-4 inhibitors at baseline, 6/10 in the LCK group discontinued these medications compared with 0/6 in the MCCR group (*p* = .005). In a 12-month trial, adults with elevated HbA1c and body weight assigned to an LCK diet had greater reductions in HbA1c, lost more weight, and reduced more medications than those instructed to follow an MCCR diet.

## Introduction

Nutritional management is an important component in the treatment of type 2 diabetes. Considerable uncertainty exists, though, about the optimal level of carbohydrate intake. Previous research suggests that an ad libitum very low-carbohydrate ketogenic diet (LCK) may improve metabolic measures in adults with type 2 diabetes^1–6^ and reduce the need for diabetes-related medications^7–10^.

We randomized adults with type 2 diabetes or prediabetes and elevated body weight to receive an LCK diet or a moderate-carbohydrate, calorie-restricted, low-fat diet (MCCR). We previously reported the initial 3-month outcomes, which showed that participants in the LCK group had significantly better glycemic control and a trend toward greater weight loss compared to participants in the MCCR group^11^. Here we report the outcomes of the trial at 6 and 12 months after baseline.

## Methods

### Study design

We conducted a parallel-group randomized (1:1) trial. The University of California, San Francisco (USCF) Institutional Review Board approved of this trial, which is registered with ClinicalTrials.gov (NCT01713764). Eligibility criteria included being aged 18 or older, overweight (body mass index (BMI) of 25 or above), with a current glycated hemoglobin (HbA_1c_) level over 6.0%. We excluded participants who were currently using insulin or taking more than three glucose-lowering agents. We obtained an informed consent from all eligible participants.

### Intervention

Participants attended 19 classes over 12 months: Twelve 2-h weekly classes, then three 2-h classes every 2 weeks, followed by four 1.5-h classes every 2 months. One group leader instructed LCK participants to eat an ad libitum very low-carbohydrate, likely ketogenic diet, by reducing their carbohydrate intake to between 20–50 g of carbohydrates (excluding fiber) a day. We gave them a goal of achieving a blood ketone (beta-hydroxybutyrate) level of between 0.5 and 3 millimolar (mmol), as measured twice a week for the first several months at home. A different group leader instructed the MCCR participants to follow an MCCR diet in which 45–50% of their calories were to be derived from carbohydrates. We also instructed them to lower their fat consumption and eat 500 fewer kilocalories (kcal) per day than their calculated maintenance needs to reduce weight.

Starting in week 6, group leaders taught participants in both groups about the importance of sleep and exercise for type 2 diabetes and encouraged them to increase both, if needed. A third group leader also taught all participants supportive behavioral adherence strategies aimed at increasing positive affect^12^ and mindful eating^13^, in order to increase intervention adherence. See our publication of the 3-month outcomes from this study for full method specifications^11^.

### Assessments

We obtained a fasting blood specimen and measured participants’ HbA_1c_, lipids, fasting glucose and insulin, and C-reactive protein. Tests were performed by a commercial CLIA-certified laboratory (Quest Diagnostics, Madison, NJ), which was unaware of the study design or assigned conditions. Study staff measured weight and blood pressure during in-person visits. We estimated insulin resistance using homeostatic model assessment (HOMA2-IR)^14^. Participants recorded their food using the Automated Self-Administered 24-h Dietary Recall (ASA24)^15^. At baseline and 3, 6, and 12 months after baseline we asked participants to report what they ate during one day.

### Statistical analysis

The statistical analyses were performed using Stata, IC software version 14.1 (StataCorp LP, College Station, TX, USA). The outcomes were continuously scaled and were analyzed with parametric statistical techniques. Several outcomes required log transformation prior to analysis and exclusion of a few influential outliers. Participants’ repeated measures outcomes were submitted to separate mixed-effects linear regression analyses with fixed effect terms comparing baseline to each of the three subsequent time points after baseline, the main effect for group, and the simple interaction effects comparing the relative change by group at each post-baseline assessment, relative to baseline. Random y-intercept terms were included to accommodate for the repeated measures experimental design. For all results involving dichotomous outcomes, we used a two-tailed Fisher exact test to assess significance. We tested the difference between groups for percent weight lost with an independent samples *t*-test for the change within each group. As this was an exploratory pilot trial, the trial was not powered.

## Results

### Recruitment and participation

We enrolled and randomized 34 participants to the LCK (*n* = 16) or MCCR (*n* = 18) group (Supplementary Fig. 1). Retention did not differ by intervention group: 12-month retention for the LCK group was 14/16 (87.5%) and 15/18 (83.3%) for the MCCR group (*p* = 1.000), with an average of 85.3% participants retained (Supplementary Table 1).

### Dietary assessment

Compared to the MCCR group, the LCK group reported consuming fewer non-fiber grams of carbohydrates (6 and 12 months), more grams of fat (6 and 12 months), and more grams of protein (12 months), but not a different number of calories per day (Table 1).

**Table 1:** Estimated marginal mean (EMM) ± 95%CI at baseline to 6 and 12 months

Outcomes	LCK group, EMM (95% CI)	MCCR, EMM (95% CI)	*P* value (interactions comparing group differences in changes relative to baseline)
HbA_1c_ (%)			
Baseline	6.6 (6.3, 6.9)	6.9 (6.6, 7.2)	
6 months	6.0 (5.7, 6.3)	6.7 (6.4, 6.9)	.001
12 months	6.1 (5.8, 6.4)	6.7 (6.4, 7.0)	.007
Body mass index (kg/m^2^)			
Baseline	35.9 (32.5, 39.2)	36.9 (33.7, 40.1)	
6 months	33.7 (30.3, 37.1)	36.0 (32.9, 39.2)	.001
12 months	33.3 (29.9, 36.7)	36.0 (32.8, 39.2)	<.001
Body weight (kg)			
Baseline	99.9 (88.4, 111.5)	97.5 (86.6, 108.3)	
6 months	93.8 (82.3, 105.3)	95.8 (84.9, 106.6)	<.001
12 months	92.0 (80.5, 103.6)	95.8 (84.9, 106.6)	<.001
Triglycerides (mg/dL)			
Baseline	102.6 (81.8, 123.4)	158.9 (128.8, 189.1)	
6 months	86.2 (68.6, 103.7)	143.2 (115.6, 170.9)	.48
12 months	92.7 (73.6, 111.7)	173.4 (138.1, 208.7)	.08
HDL cholesterol (mg/dL)			
Baseline	48.4 (42.6, 54.2)	45.8 (40.6, 51.0)	
6 months	51.9 (45.7, 58.2)	48.1 (42.5, 53.6)	.58
12 months	53.3 (46.8, 59.8)	48.9 (43.3, 54.5)	.45
LDL cholesterol (mg/dL)			
baseline	88.7 (76.3, 101.1)	98.1 (86.4, 109.8)	
6 months	97.9 (85.4, 110.5)	88.1 (76.0, 100.1)	.003
12 months	95.6 (82.3, 108.9)	96.1 (83.7, 108.5)	.20
Triglycerides (mg/dL)/HDL cholesterol (mg/dL)			
baseline	2.2 (1.7, 2.7)	3.5 (2.7, 4.2)	
6 months	1.7 (1.3, 2.1)	3.0 (2.4, 3.7)	.18
12 months	1.7 (1.3, 2.1)	3.6 (2.8, 4.5)	.022
C-reactive protein (mg/dL)			
Baseline	4.2 (2.1, 6.4)	3.3 (1.7, 4.9)	
6 months	2.9 (1.4, 4.5)	2.5 (1.3, 3.6)	.69
12 months	3.0 (1.5, 4.6)	2.3 (1.2, 3.4)	.85
Fasting insulin (µIU/mL)			
Baseline	8.7 (6.0, 11.3)	8.9 (6.4, 11.5)	
6 months	8.9 (6.2, 11.6)	11.9 (8.4, 15.3)	.09
12 months	9.1 (6.3, 12.0)	10.1 (7.1, 13.0)	.66
HOMA2-IR			
Baseline	1.0 (0.7, 1.4)	1.1 (0.8, 1.5)	
6 months	1.2 (0.8, 1.5)	1.4 (1.0, 1.9)	.38
12 months	1.0 (0.7, 1.3)	1.2 (0.9, 1.6)	.51
Diastolic blood pressure (mm Hg)			
Baseline	77.1 (74.0, 80.3)	81.1 (78.2, 84.1)	
6 months	77.1 (74.0, 80.1)	80.8 (77.9, 83.7)	.90
12 months	75.6 (72.5, 78.8)	78.4 (75.5, 81.4)	.57
Systolic blood pressure (mm Hg)			
Baseline	127.1 (121.9, 132.3)	129.2 (124.6, 133.7)	
6 months	130.7 (125.7, 135.7)	130.4 (125.6, 135.1)	.47
12 months	130.3 (125.2, 135.4)	127.5 (122.7, 132.4)	.15
Total kilocalories			
Baseline	1954.1 (1617.7, 2290.5)	2063.1 (1733.5, 2392.7)	
6 months	1589.4 (1315.1, 1863.7)	1483.5 (1236.7, 1730.3)	.33
12 months	1534.8 (1255.5, 1814.0)	1681.1 (1387.4, 1974.9)	.78
Total grams of non-fiber carbohydrates			
Baseline	176.2 (143.9, 208.6)	184.4 (152.7, 216.2)	
6 months	44.1 (27.4, 60.8)	160.7 (131.0, 190.4)	<.001
12 months	73.7 (51.5, 96.0)	149.8 (119.4, 180.2)	.002
Total grams of fat			
Baseline	79.2 (59.8, 98.6)	86.3 (66.8, 105.8)	
6 months	101.4 (76.5, 126.3)	55.8 (42.6, 69.0)	.001
12 months	105.4 (79.4, 131.3)	75.4 (56.5, 94.2)	.037
Total grams of protein			
Baseline	82.7 (65.1, 100.4)	91.4 (72.6, 110.3)	
6 months	92.2 (72.4, 112.0)	82.7 (65.9, 99.5)	.12
12 months	97.6 (76.3, 118.9)	68.8 (53.8, 83.9)	.002

Data are estimated marginal means and 95% confidence intervals by linear mixed-effects model analysis

### HbA_1c_

**Fig. 1:**
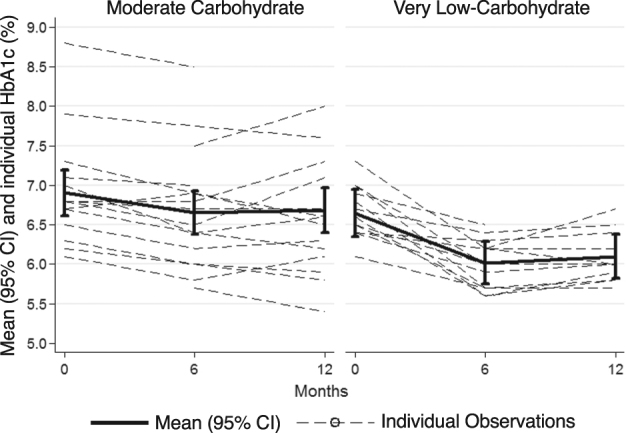
Mean and individual HbA_1c_ for the two groups at baseline and at 6 and 12 months Bars represent standard 95% confidence intervals of the mean. Dashed lines reflect individual participant observations; darker lines represent each group mean

At 12 months, participants in the LCK group reduced their HbA_1c_ levels more than participants in MCCR group (Table 1, Fig. 1). In the LCK versus the MCCR group, at both 6 and 12 months, more than twice the percentage of participants who began with an HbA_1c_ at or above 6.5%, the cutoff for type 2 diabetes, ended below this level. However, this result was only significant at 6 months (Supplementary Table 2).

### Body weight and other health outcomes

At 12 months, participants in the LCK group lost more weight and lowered their BMI more than participants in the MCCR group (Table 1, Supplementary Fig. 2). On average, at 12 months participants in the LCK group lost 8.3% of body weight, whereas the MCCR group lost 3.8% (Supplementary Table 2).

At 6 months, LDL cholesterol increased more in the LCK group compared to the MCCR group, although at 12 months the groups no longer significantly differed (Table 1). At 6 months, change in the ratio of triglycerides to HDL cholesterol did not significantly differ across groups, although at 12 months this ratio had decreased more in the LCK group compared to the MCCR group (Table 1). Other biological outcomes did not differ significantly across groups (Table 1).

### Diabetes medications

Participants in the LCK group reduced their use of some diabetes-related medications more than participants in the MCCR group. Of ten participants who reported taking sulfonylureas or dipeptidyl peptidase-4 inhibitors before the intervention, all six participants assigned to the LCK group discontinued these medications by 12 months post-baseline (at the request of the study physicians, based on the study protocol), compared with none of the four participants in the MCCR group (*p* = .005, Fischer’s exact test). Two participants in the MCCR group began taking these medications, whereas no participants in the LCK group did so. Of 22 participants who reported taking metformin before the intervention, 3/10 in the in the LCK group discontinued the medication, compared with 0/12 in the MCCR group (*p* = .08, Fisher’s exact test). In addition, in the LCK group 1 person increased their dose of metformin and in the MCCR group 2 people decreased their metformin, with none of these changes being significantly different between groups (*p* > .3).

## Discussion

Twelve months after baseline, participants in the LCK group evidenced greater reductions in each HbA_1c_ and weight than did participants in the MCCR group. In addition, the greater reductions in HbA_1c_ in the LCK group occurred despite greater reductions in glucose-lowering medications. A strength of our trial was that few participants dropped out after 12 months. However, the ability to generalize from this study is limited by its relatively small size, which did not allow us to perform subgroup analyses.

At 6 months, we noted an increase in LDL cholesterol in the LCK group compared to the MCCR group. This difference was no longer significant at 12 months. This may raise some concerns about the long-term effects of such a diet on cardiovascular disease. Recent research suggests that the correlation of LDL to cardiovascular risk varies based on particle size^16, 17^, and that low-carbohydrate ketogenic diets tend to increase LDL particle size^18^, which suggests that the increase in total LDL may not be accompanied by increased cardiovascular risk. However, we did find that the ratio of triglycerides to HDL, which predicts coronary disease^19^, decreased in the LCK group compared to the MCCR group, suggesting that the very low-carbohydrate ketogenic diet may have certain benefits on lipid profiles. In addition, no participants experienced diabetic ketoacidosis, a condition that occurs when ketone production is unregulated, with blood ketone levels surpassing 25 mM, with concurrent high blood glucose levels (above 250 mg/dL), an unlikely scenario in this research^20^.

Our trial had good retention and fair dietary adherence, which may have been due to the novel supportive psychological strategies (positive affect and mindful eating). Future trials could explicitly test the hypothesis that these strategies may improve retention and adherence rates. Moreover, because both groups also received lifestyle recommendations (physical activity and sleep), we are limited in understanding how the diets may have influenced outcomes independent of the other suggested changes. In future trials, if researchers randomized participants to different combinations of lifestyle and psychological recommendations, this could clarify and optimize the most helpful adjunctive advice and support.

The results suggest that adults with prediabetes or noninsulin-dependent type 2 diabetes may be able to improve glycemic control with less medication by following an ad libitum very low-carbohydrate ketogenic diet compared to a moderate-carbohydrate, calorie-restricted low-fat diet. Additional research should examine both clinical outcomes and adherence beyond 12 months.

## Electronic supplementary material

Supplementary File: Figure Legends, Table

Supplementary Figure 1

Supplementary Figure 2
