# Supplementary material for: Twelve-month outcomes of a randomized trial of a moderate-carbohydrate versus very low-carbohydrate diet in overweight adults with type 2 diabetes mellitus or prediabetes
Source: Nutr Diabetes. 2017 Dec 21;7(12):304. doi: 10.1038/s41387-017-0006-9 (PMC5865541; doi:10.1038/s41387-017-0006-9)
Supplement: Supplementary file 1 — Supplementary File: Figure Legends, Table [file 41387_2017_6_MOESM1_ESM.docx]

Supplementary Figure 1.

Tile: Study participant ﬂowchart.

Supplementary Figure 2.

Title: Mean and individual body weight (in kilograms) for the two groups at baseline and at 6 and 12 months.

Legend: Bars represent 95% confidence intervals of the mean. Dashed lines reflect individual participants; darker lines represent each group mean.

Supplementary Information Table 1. Baseline participant characteristics.

|  | LCK group (*n*=16) | MCCR group (*n*=18) |
| --- | --- | --- |
| Sex, *n* (%) |  |  |
| Male | 7 (44) | 2 (11) |
| Female | 9 (56) | 16 (89) |
| Age, mean (SD), years | 64.8 (7.7) | 55.1 (13.5) |
| Race/Ethnicity, *n* (%) |  |  |
| Asian/Pacific Islander | 1 (6) | 4 (22) |
| Black | 1 (6) | 1 (6) |
| White | 13 (81) | 11 (61) |
| Latino/a | 1 (6) | 2 (11) |
| Duration of type 2 diabetes or prediabetes (years, mean (SD) | 7.6 (7.7) | 6.4 (4.9) |
| Baseline HbA_1c_ started at or above 6.5%, *n* (%) | 9 (56.3) | 13 (72.2) |

Information for entire sample, regardless of follow-up data.

Supplementary Information Table 2. Percent of people meeting HbA_1c_ and weight change thresholds and percent weight loss.

|  | LCK group | MCCR group | *P* value |
| --- | --- | --- | --- |
| Participants whose HbA_1c_ started at or above 6.5% and finished below 6.5%, ratio (percent) |  |  |  |
| 6 months | 8/9 (88.9%) | 5/13 (38.5%) | .03 |
| 12 months | 7/9 (77.8%) | 4/13 (30.8%) | .08 |
| Weight (% of initial weight), mean (SD) |  |  |  |
| 6 months | -7.1 (4.5) | -2.7 (5.4) | .02 |
| 12 months | -8.3 (5.8) | -3.8 (6.0) | .05 |
| Participants achieving a 5% weight loss, ratio (percent) |  |  |  |
| 6 months | 10/15 (66.7%) | 5/16 (31.25%) | .08 |
| 12 months | 10/14 (71.4%) | 5/15 (33.3%) | .07 |

The difference between groups for percent weight lost tested with an independent samples *t*-test for the change within each group. For results involving ratios, we used a two-tailed Fisher exact test to assess significance.
