# Supplementary material for: Twelve-month outcomes of a randomized trial of a moderate-carbohydrate versus very low-carbohydrate diet in overweight adults with type 2 diabetes mellitus or prediabetes
Source: Nutr Diabetes. 2017 Dec 21;7(12):304. doi: 10.1038/s41387-017-0006-9 (PMC5865541; doi:10.1038/s41387-017-0006-9)
Supplement: Supplementary file 2 — Supplementary Figure 1 [file 41387_2017_6_MOESM2_ESM.pdf]

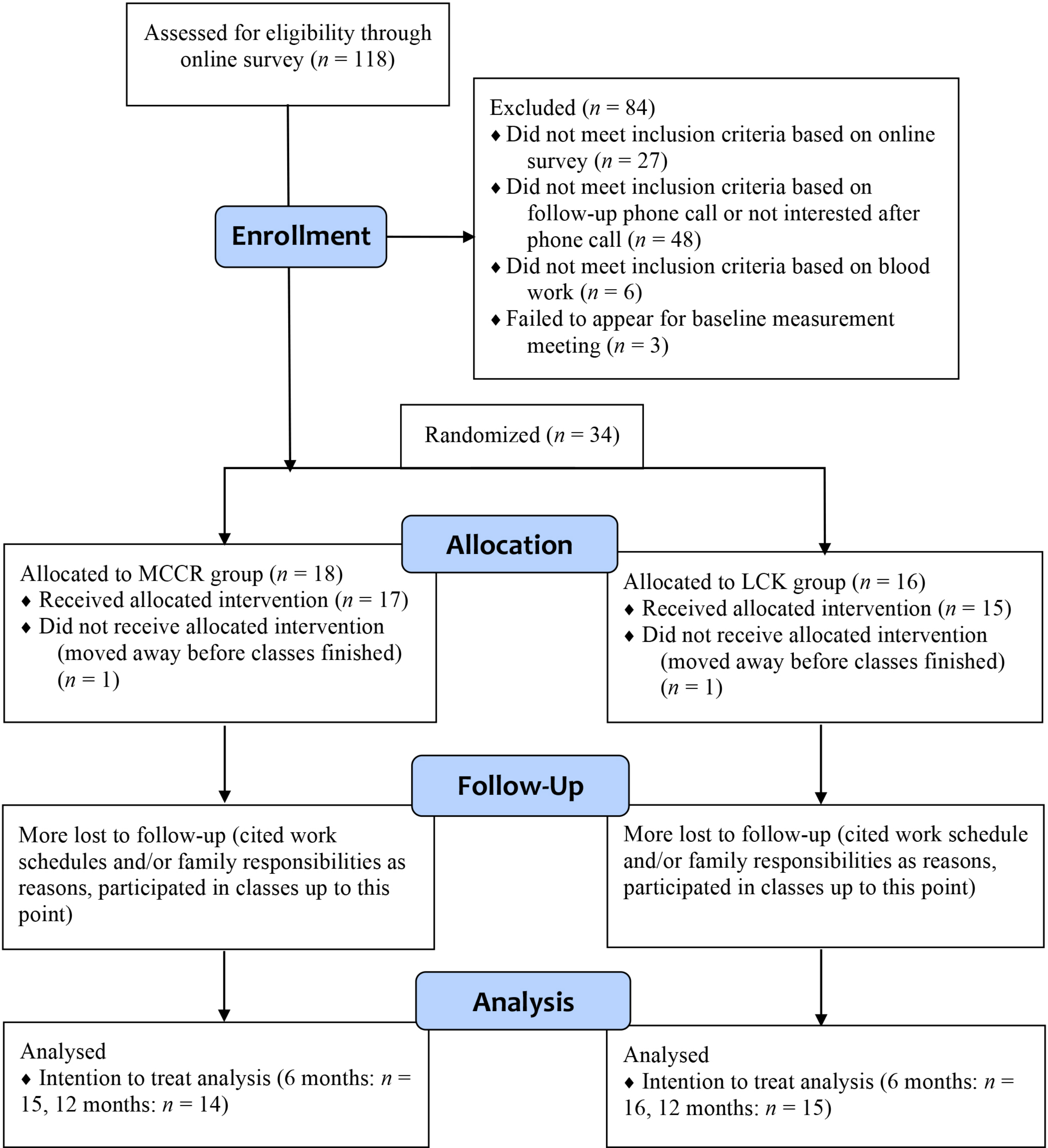
Assessed for eligibility through online survey (n = 118)
Enrollment
Excluded (n = 84)
♦ Did not meet inclusion criteria based on online survey (n = 27)
♦ Did not meet inclusion criteria based on follow-up phone call or not interested after phone call (n = 48)
♦ Did not meet inclusion criteria based on blood work (n = 6)
♦ Failed to appear for baseline measurement meeting (n = 3)
Randomized (n = 34)
Allocation
Allocated to MCCR group (n = 18)
♦ Received allocated intervention (n = 17)
♦ Did not receive allocated intervention (moved away before classes finished) (n = 1)
Allocated to LCK group (n = 16)
♦ Received allocated intervention (n = 15)
♦ Did not receive allocated intervention (moved away before classes finished) (n = 1)
Follow-Up
More lost to follow-up (cited work schedules and/or family responsibilities as reasons, participated in classes up to this point)
More lost to follow-up (cited work schedule and/or family responsibilities as reasons, participated in classes up to this point)
Analysis
Analysed
♦ Intention to treat analysis (6 months: n = 15, 12 months: n = 14)
Analysed
♦ Intention to treat analysis (6 months: n = 16, 12 months: n = 15)
