# Supplementary figures and images for: Twelve-month outcomes of a randomized trial of a moderate-carbohydrate versus very low-carbohydrate diet in overweight adults with type 2 diabetes mellitus or prediabetes
Source: Nutr Diabetes. 2017 Dec 21;7(12):304. doi: 10.1038/s41387-017-0006-9 (PMC5865541; doi:10.1038/s41387-017-0006-9)

Moderate Carbohydrate

Very Low-Carbohydrate

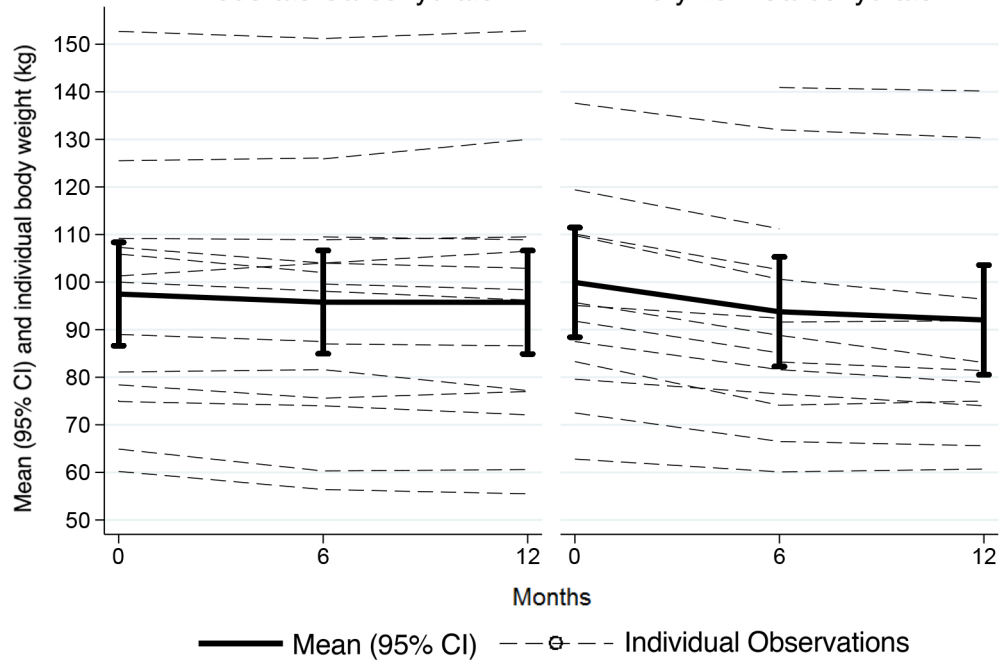

Supplement: Supplementary file 3 — Supplementary Figure 2 [file 41387_2017_6_MOESM3_ESM.pdf]
